# Sea Ice Biogeochemistry: A Guide for Modellers

**DOI:** 10.1371/journal.pone.0089217

**Published:** 2014-02-25

**Authors:** Letizia Tedesco, Marcello Vichi

**Affiliations:** 1 Marine Research Centre, Finnish Environment Institute, Helsinki, Finland; 2 Istituto Nazionale di Geofisica e Vulcanologia, Bologna, Italy; 3 Centro Euro-Mediterraneo sui Cambiamenti Climatici, Bologna, Italy; University of Aveiro, Portugal

## Abstract

Sea ice is a fundamental component of the climate system and plays a key role in polar trophic food webs. Nonetheless sea ice biogeochemical dynamics at large temporal and spatial scales are still rarely described. Numerical models may potentially contribute integrating among sparse observations, but available models of sea ice biogeochemistry are still scarce, whether their relevance for properly describing the current and future state of the polar oceans has been recently addressed. A general methodology to develop a sea ice biogeochemical model is presented, deriving it from an existing validated model application by extension of generic pelagic biogeochemistry model parameterizations. The described methodology is flexible and considers different levels of ecosystem complexity and vertical representation, while adopting a strategy of coupling that ensures mass conservation. We show how to apply this methodology step by step by building an intermediate complexity model from a published realistic application and applying it to analyze theoretically a typical season of first-year sea ice in the Arctic, the one currently needing the most urgent understanding. The aim is to (1) introduce sea ice biogeochemistry and address its relevance to ocean modelers of polar regions, supporting them in adding a new sea ice component to their modelling framework for a more adequate representation of the sea ice-covered ocean ecosystem as a whole, and (2) extend our knowledge on the relevant controlling factors of sea ice algal production, showing that beyond the light and nutrient availability, the duration of the sea ice season may play a key-role shaping the algal production during the on going and upcoming projected changes.

## Introduction

Sea ice plays a key role in the climate system [1], mainly due to the albedo positive feedback [2] and to the amplified climate changes undergoing in sea ice-covered regions [3]. In parallel, the ice-associated (sympagic) biology has a key role in winter ecology of ice-covered waters [4]. During winter months sea ice algae are essential to overwintering zooplankton, being the only food source available [5]. Most of the regions seasonally covered by sea ice are the most productive of the oceans, with a shorter production season but more intense algal blooms (e.g. [6]). The highest algal cell and chlorophyll (Chl) concentrations of any aquatic environment has been found in sea ice [7]. Besides the qualitative and quantitative relevance of sea ice algae, recent works have highlighted the importance of sea ice for e.g. dimethyl sulfide production [8], source/sink of CO_2_ [9], bioaccumulation of iron [10], and enhanced CaCO_3_ precipitation [11]. Indirectly, the presence of sea ice also affects the pelagic dynamics: under-ice phytoplankton blooms can be massive when compared to adjacent open water areas [12].

While biogeochemical models of the pelagic ecosystem are commonly developed in the Arctic Ocen [13] and in the Southern Ocean [14], and more recently used to assess potential changes in the ecosystem dynamics under future climate change scenarios [15], the same cannot be said of sea ice biogeochemical models, mostly excluded in large-scale studies except in rare cases [16]–[19]. Ignoring to include the sea ice biogeochemical component in modelling studies of polar oceans implies neglecting the quantitative and qualitative importance that we currently know sea ice biogeochemistry holds.

Sea ice has been long time considered an impermeable layer between the ocean and the atmosphere, and a rather thin layer when compared with the depths of the oceans. More recently the biogeochemical importance of sea ice in global biogeochemical cycles has been reviewed [20] and large scale Chl data collection has been organized in the Southern Ocean [21], showing the large spatial and temporal patchiness of the observations. Sea ice sampling presents several difficulties: weather conditions often limit data collection, while sampling methods are either time consuming and/or expensive. Comprehensive modelling studies may thus be the most suitable method to integrate among sparse observations, contributing to the understanding of the role that sea ice biogeochemistry plays in the past, present and future state of the polar oceans ([20]–[24]).

A major aim of this paper is to formulate a theoretical background for the construction of applicative models of sea ice ecosystems. The presented conceptual study stems from a previous application that was thoroughly tested against observations [25]. The model used in [25] demonstrated to satisfactorily capture the specific environmental features of a typical Arctic site in the Greenland Sea as well as the more variable conditions in a Baltic Sea location. By distilling from this previously validated model the theoretical relationships of the dependence on external forcing functions, we aim at making more evident the major factors controlling sea ice algae dynamics. We develop a methodology to build sea ice biogeochemical models starting from a pelagic biogeochemical model and including the key functional types found in sea ice. This methodology is highly flexible and can be applied to any existing ocean model, despite its resolution and complexity. Our current knowledge and application of one-dimensional coupled sea ice physical-biogeochemical models span from prescribed sea ice physical properties to mushy layer theory [23], from simple nutrient-phytoplankton-detritus (NPD) models to stoichiometrically-flexible multiple Plankton Functional Type models [25] (see Table 1 for a list of sea ice biogeochemical models). Since the aim of this work is to encourage modelers of the marine ecosystem to include a new component to their modelling framework wherever and whenever sea ice is part of it, we chose to apply our general methodology to a model of intermediate complexity, yet computationally feasible for large-scale coupled configurations, but with realistic biological and physical descriptors.

**Table 1:** List of sea ice biogeochemical models and their components, revised and extended after [20].

Reference	N. of groups	Constit.	Layers	Ice-ocean fluxes	Ocean
Arrigo et al, 1993 [40]	3N-1P	n p s	ML	Diffusion	n.a.
Arrigo et al., 1997[58]	3N-1P	n p s	1L static	Diffusion	n.a.
Lavoie et al., 2005 [37]	1N-1P	s	1L static	Diffusion	n.a.
Nishi and Tabeta, 2005 [38]	2N-1P-1Z-2D	n s c l	1L static	Diffusion, Convection	1D
Jin et al., 2006 [36]	3N-1P	n s	1L static	Diffusion	1D
Lavoie et al., 2009 [59]	1N-1P	s	1L static	Diffusion	1D
Tedesco et al., 2010 [25]; Tedesco et al., 2012 [28]	4N-2P-2D	n p s l c	1L dynamic	Growth/melt	1D
Vancoppenolle et al., 2010[39]	1N	s	ML	Growth/melt, brine transport	n.a.
Pogson et al., 2011 [60]	1N-1P	s	ML	Diffusion	n.a.
Deal et al., 2011 [16]; Jin et al., 2012 [17]	3N-1P	n s	1L static	Diffusion	3D
Sibert et al, 2011 [19]	1N-1P-1Z	n	1L static	Turbolent	3D
Elliott et al., 2012 [61]	3N-1P-1Z-1D	n s l c f	1L static	Diffusion	3D
Saenz and Arrigo, 2012 [62]	3N-1P	n p s	ML	Desalination	n.a.
Rubao et al., 2013 [15]	3N-1P	n s f	1L static	Diffusion	3D

N  = nutrient; P  =  algae; D  =  detritus; Z  =  fauna; n  =  nitrogen; p  =  phosphorous; s  =  silicon; l  =  chlorophyll; c  =  carbon; f =  sulfur; ML  =  multi-layer; 1L  =  1 layer.

Among the ice-covered oceans, the Arctic is the one facing the most dramatic changes: the sea ice pack has decreased by more than 40% in the last three decades [26], though modulated by large variability. Between the 2012 new summer minimum and the 2013 winter maximum, the Arctic Ocean has registered the largest increase in ice extent in the satellite records (NOAA press release, April 2, 2013). First-year ice is rapidly replacing multi-year ice and projections show that the Seasonal Ice Zone might cover the entire Arctic as early as the 2020s [27]. It is therefore needed to foster the development of models on the study of the controlling factors of nutrients and algae in a typical season of Arctic first-year ice, as the first-year ice is the most common type of ice that we are expected to encounter in the near future. We show how even a simple conceptual and numerical model exercise can help to further extend our knowledge on the limiting factors controlling sea ice algae growth, and we will do it by proposing specific ecological indicators. We will analyze in particular the different effect of some selected physical (i.e. snow cover) and biological (i.e. nutrient availability) controlling factors on the growth of sea ice algae in the current state and considering a shortening of the ice seasons. We will finally show that the benefits of modelling coupled sea ice-pelagic biogeochemistry are manifold: it allows to simulate all-year-around biogeochemistry without the need of seasonal initial conditions, it will ensure a more adequate representation of the ecosystem as a whole with a more realistic representation of the oceanic spring bloom [28] and always ensuring a strict mass conservation, the only but extremely important law that marine ecosystem models have.

## Methods: Setting the Stage

### A conceptual sea ice ecosystem

The limited number of biogeochemical models implemented for the sea ice ecosystem with respect to those implemented for the adjacent pelagic ecosystem is generally attributed to large uncertainties in sea ice biogeochemical processes. While this may be true for specific processes that are still difficult to quantify in a general framework (e.g. calcium carbonate precipitation, [11]), most main physiological and ecological processes that occur in sea ice are the same as in seawater: e.g. photosynthesis, respiration, exudation, remineralization.

Sea ice is made of a pure solid matrix, liquid saline brines, gas bubbles and impurities. The biological community that is found as sea ice grows is composed of bacteria, microalgae and heterotrophic protists that live in brine pockets and channels, the liquid saline fraction of sea ice. Sea ice is characterized by steep gradients in temperature, salinity, light and space (brines) availability, and those are the major physical constrains to microalgae's growth, making the sea ice bottom a more similar habitat to that of seawater, thus more suitable for the biological community. On the other hand, light is strongly attenuated from surface to bottom sea ice, especially if sea ice is snow-covered. Thus photosynthetic organisms require adaptation/acclimation to low temperature, high salinity and/or low light intensities in order to survive and/or bloom in sea ice. In general, rates of physiological processes such as photosynthesis increase with temperature up to some point and this is true also for polar species [29]. The Q_10_, a measure of the rate of change of algal growth as a consequence of increasing the temperature by 10 degree Celsius, originally proposed by [30], is often used to quantify these metabolic rates in models. Ice algae have been found to have a Q_10_ ranging between 1.0 and 6.0 [31], indicating high potential of acclimation [32]. In addition to temperature, as salinity diverges from sea water values, growth rates, photosynthetic efficiency and capacity of sea ice algae are reduced [33]. Sea ice algae living under several meters of ice and snow have shown to have some of the most extreme low-light adaptation [34], while low-light acclimation is accomplished by e.g. an increase in photosynthetic efficiency [35] and in photosynthetic units per cell [14].

**Figure 1:**
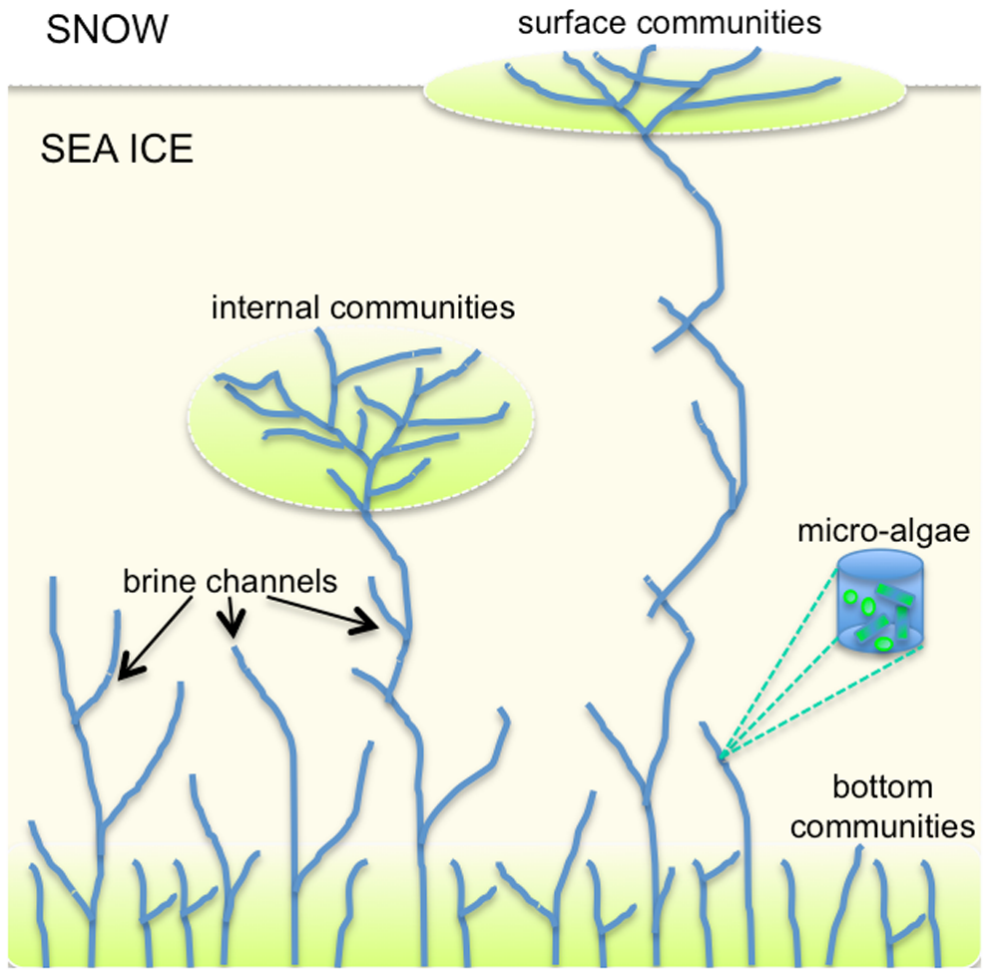
Schematic distribution of ice algae in sea ice. Sea ice algae are generally found at the bottom of sea ice, where temperature and salinity are more similar to those of seawater, and where light is the most likely limiting factor to growth. If snow ice is formed, seawater might reach the sea ice/snow interface and some communities develop at the surface. During rafting and ridging, occasionally seawater can be placed at intermediate depths and the internal communities grow.

Dissolved nutrients are found in the brines, thus their concentration is usually higher at the bottom of sea ice than at the surface. Occasionally, heavy snow loads lower the freeboard bringing seawater at the interface between snow and ice where surface communities might flourish. Similarly, during rafting events, some seawater can be placed in the interior of sea ice where the internal communities can develop. Thus, depending on their ability to adapt/acclimate, photosynthetic organisms are differently distributed in the bottom, interior and surface of sea ice (Fig. 1). Those differences can be addressed by the choice of different functional types of organisms or by different parameter values. However, whatever diversity is found in sea ice, the same diversity characterizes the ocean underneath, simply by considering mass conservation. Thus, independently of the ecosystem complexity chosen for modelling the pelagic ecosystem, a similar level of complexity should be used to model the sea ice community, and this is true for simple and for more comprehensive models: the numbers of functional groups in sea ice must be the same or smaller than in seawater and the biogeochemical processes that characterizes the chosen functional groups will be the same in both environments. This is easily done when coupling the two components and letting each chemical functional group (or a reduced number) found in seawater to be able to be transferred to sea ice and viceversa, as we will see in detail in the following sections.

### Vertical representation

**Figure 2:**
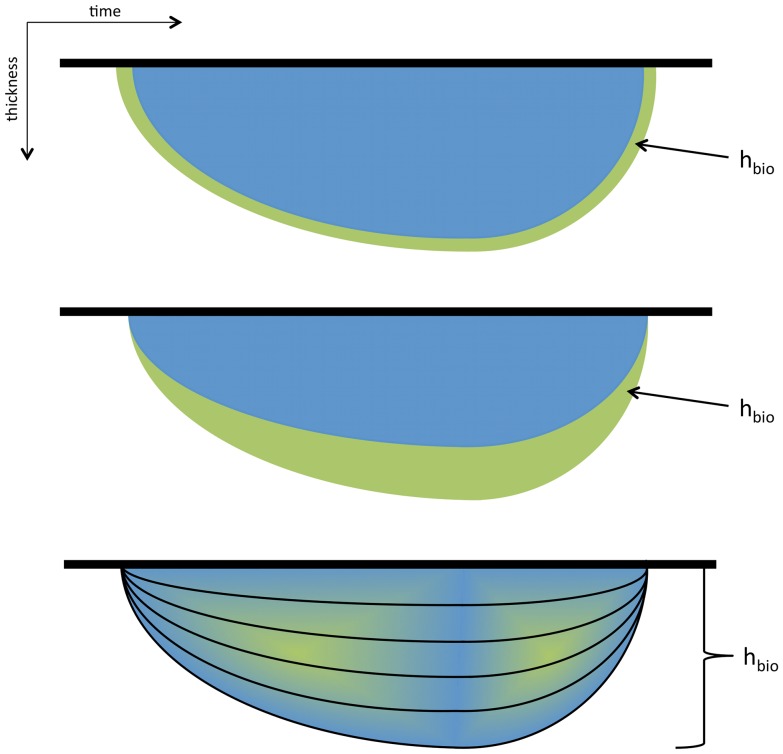
The choice of the vertical representation of the sea ice biogeochemical model. A comparison between three different layer models: a) skeletal layer: a bottom layer of prescribed thickness; b) Biologically-Active Layer (BAL): the bottom sea ice layer that is permeable (relative brine volumes larger than 5%) during the entire ice season; 3) multi-layer: a prescribed number of ice layers of the same thickness.

Biogeochemical reactions occur in brines, which are the liquid fraction of sea ice. Brines are not uniformly distributed in sea ice. In an ideal sea ice season, during which first-year ice does not deform and does not depress, sea ice brines are constantly larger at the bottom - and are likely to exceed the permeability threshold - and become smaller towards the surface. In this ideal ice season the development of the biological community is only at the sea ice bottom and a 2-layers model may be considered sufficient to realistically represent the biological and non-biological fraction of sea ice. The simplest approach is to consider a single sea ice layer of constant thickness where biology is active (Fig. 2a), as for example the lowermost 0.02 m of multiyear Arctic sea ice [36]–[38].

This simple method tends to underestimate primary production in first-year ice, as for example shown in [25]. A possible solution to overcome this problem yet maintaining a single model layer is to compute the biologically-active fraction of sea ice as function of sea ice permeability controlled by the vertical distribution of sea ice temperature and salinity. The Biologically-Active Layer (BAL, [25], Fig. 2b) is that part of sea ice where the liquid fraction (brines) are interconnected (relative volume larger than 5 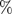) and where the biological community may develop, in contrast to the rest of the ice where brines are not connected and the biological community is less likely to survive.

In other cases, sea ice can depress and seawater can flood due to snow-ice formation, and deform due to converging or diverging of ice floes. Those are typical features of Antarctic sea ice. In such conditions brines may be temporarily large also at the surface or at intermediate depths, allowing the development of the so-called surface and internal communities, respectively, as described in Fig. 1. In these conditions a detailed vertical resolution is relevant and a multiple layer model (Fig. 2c) such as that of [39] has been shown to reproduce the process. It is important in this last case to consider that the possibility to have interior and surface communities may require a further addition of physiological traits, which may lead to a necessary increase of complexity within the sea ice ecosystem and hence the pelagic ecosystem.

### Coupling the sea ice and the pelagic systems

**Figure 3:**
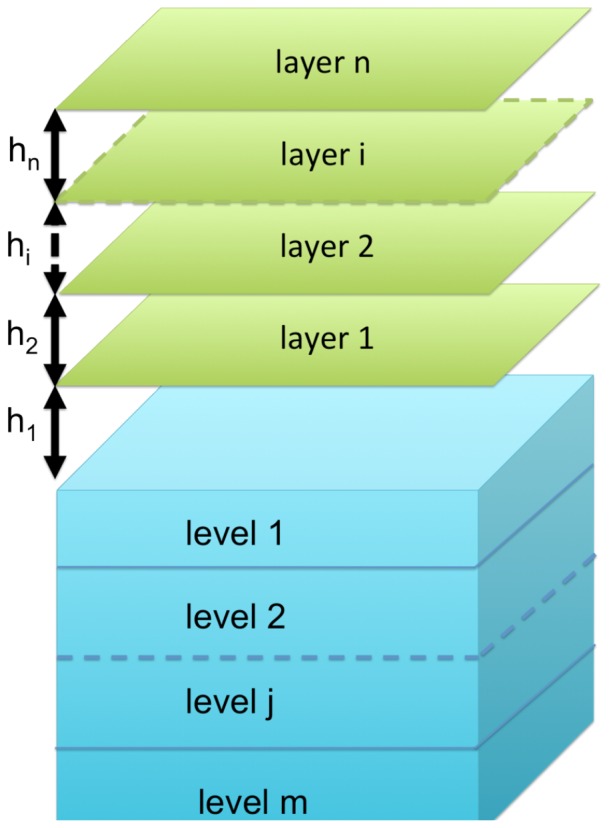
Coupling structure between a level model of the ocean with a layer model of sea ice. The coupling is done between the bottom sea ice layer n1 and the first ocean level m1, independently of the number of layers n and levels m of the model.

While sea ice and seawater are certainly characterized by similar biogeochemical processes, there are relevant physical differences between the two systems. In the ocean, particles move in all directions in space, while in sea ice particles are trapped in a semi-solid matrix and organisms are distributed in the available volume. Because the volume of brines changes with time, sea ice models are better described with one or more layers of variable thickness, where only the last one is connected with the underlying ocean model. Fig. 3 presents a simplified representation of the physical interface between a sea ice model and a typical ocean model discretized in terms of vertical levels. For completeness, we note that also the ocean model may be expressed in terms of layers of variable depth as in the case of an isopycnal model.

One dimensional (vertical) sea ice algae models have represented the coupling and the boundary fluxes at the ice-ocean interface in several ways. The more intuitive approaches are to prescribe a constant diffusivity value at the sea ice-ocean interface [37], [38] or to impose empirical functions of brine volume flux [36], [40]. A more dynamical representation may involve the computation of prognostic fluxes as a function of sea ice growth/melt velocities [25], [41] or the inclusion of mushy layer theory concepts [39], [42]. In any of these cases, the most important constraint is mass conservation. Due to the seasonal nature of first-year ice, initial concentrations of sea ice variables will only be due to the ocean boundary flux with the seawater counterparts and sea ice must be totally emptied before complete melting.

## Simulation of a Typical Sea Ice Season

### Forcing functions

Based on a published time series [43] and on previous model simulations [25], [28], [44], we designed an idealized typical season of first-year ice in the low-latitude Arctic. The proposed time evolution of the sea ice boundary conditions were linearized as much as possible to minimize the noise due to forcing and therefore highlight the major controlling factors for algal growth.

**Figure 4:**
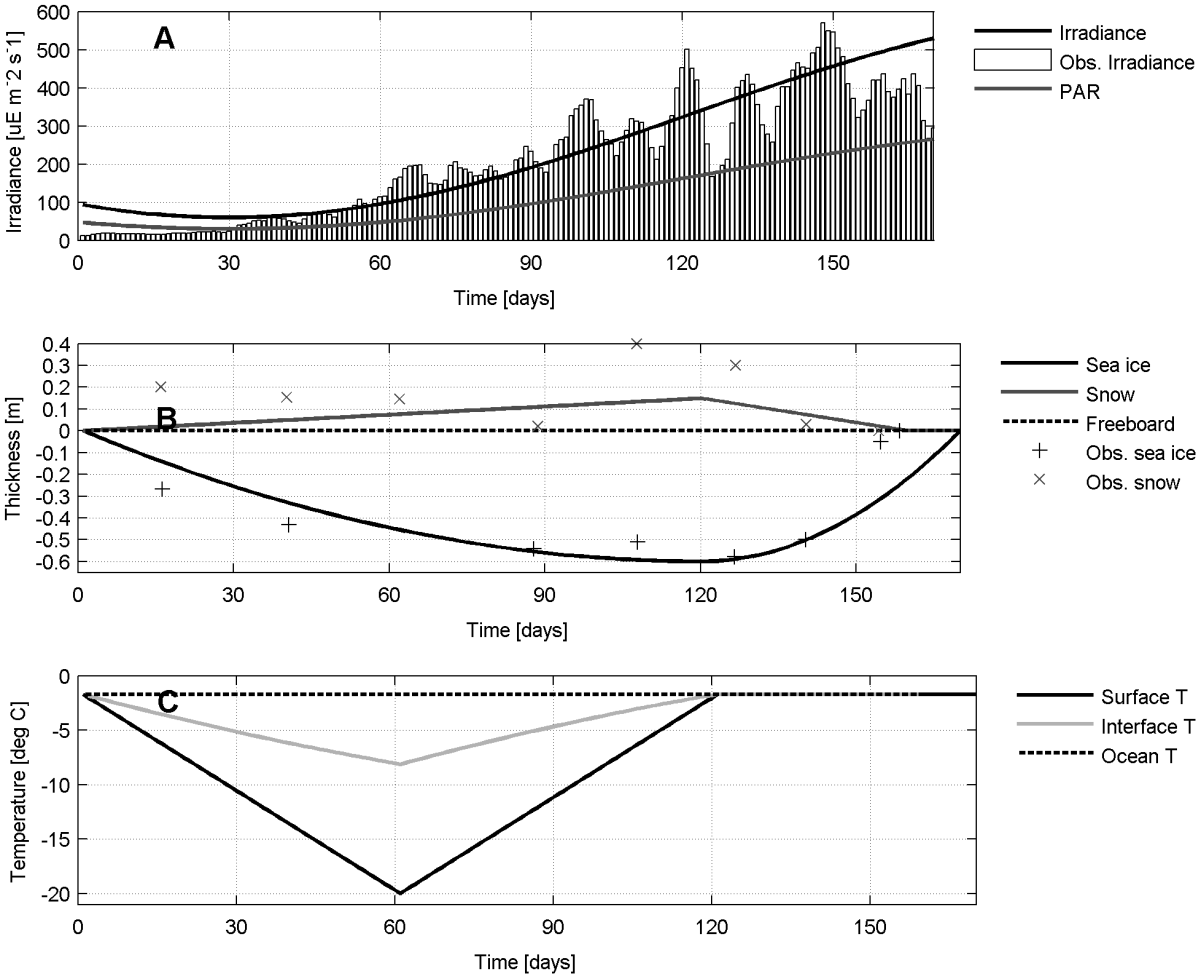
Model set up. Prescribed properties (lines) of a typical season of first-year ice in the low Arctic based on the observations (markers) reported by [43]: A) irradiance and Photosynthetic Available Radiation (PAR), B) snow and ice thicknesses, C) surface, snow/sea ice interface and ocean temperatures.

Model simulations are ideally located at 65°N, and surface irradiance values range sinusoidally between 60 and 600 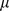
E m^−2^ s^−1^ as in [43] (Fig. 4A). Seawater salinity is fixed at 32 and seawater freezing temperature is −1.728°C. Sea ice is prescribed to grow from day 1 (i.e. December, 1st) to day 120 to a maximum thickness of 0.6 m. At day 121 sea ice starts melting and the ocean is ice-free by day 170 (i.e. May, 20th). A cubic function is used to simulate the reduced sea ice growth rate as sea ice thickens and ages with similar dynamics as in [43] (Fig. 4B). Snow thickness is generally highly variable in coastal locations as found in [43] (Fig. 4B). The high frequency variability and small spatial scales are likely to affect the local behaviour of the sea ice ecosystem and therefore the choice of an idealized forcing function representing this latitude is more difficult. We chose to use a simple linear function for snow accumulation and melt and that resembles the mean snow thickness that was measured in [43]: snow accumulates on sea ice from day 1 until day 120 and reaches a maximum thickness of 0.10 m, then it melts completely by day 160 (Fig. 4B). Seawater flooding does not occur since the ratio between snow and ice thicknesses never exceeds 1:3 (e.g.[43]), assuming an average density of 300 kg m^−3^ for snow, 900 kg m^−3^ for sea ice and 1000 kg m^−3^ for seawater. The surface temperature linearly decreases from freezing temperature on day 1 to −20°C on day 60, then linearly increases until day 120 when it reaches again the freezing point. The surface temperature is then fixed at the freezing point until the ocean is ice-free (Fig. 4C). For sake of simplicity, sea ice is isosaline (3.0) during the whole ice season.

### Model construction

The steps to be taken in the development of the sea ice biological model are:

- the choice of the vertical representation
- the choice of the ecosystem complexity
- the coupling with the ocean.

The choice of the model vertical representation must be made first, as this will determine the other steps. As our simulation considers undeformed first-year ice with no flooding event, sea ice communities will be growing only at the bottom. As a compromise between single and multiple layer models we used the approach of [25], which defines a dynamically-varying bottom layer (the Biologically-Active Layer BAL, h*_bio_* of Fig. 2b). This two-layer model allows to represent the abiotic fraction of sea ice and the permeable part characterized by a relative brine volume larger than 5%.

**Figure 5:**
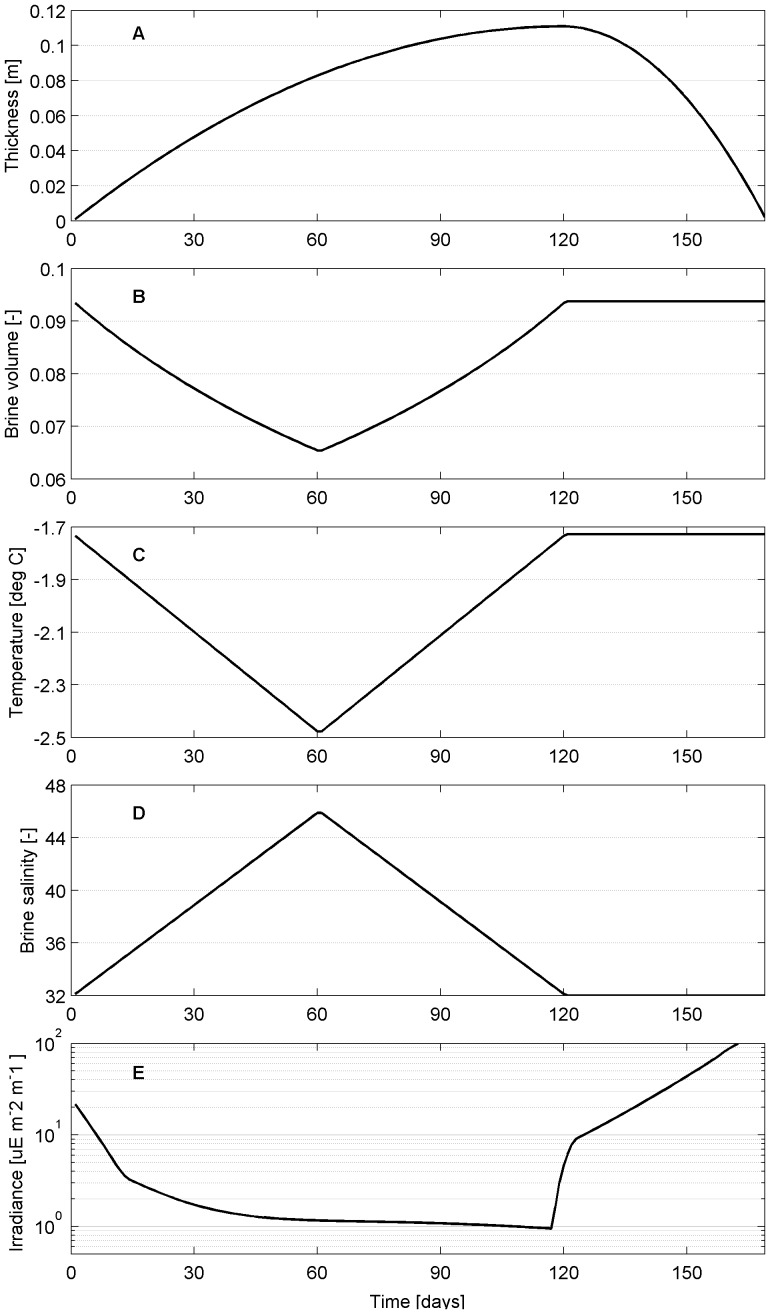
BAL properties along a typical season of first-year ice in the low Arctic. A) thickness, B) relative brine volume, C) sea ice and brines temperature, D) brines salinity, E) average PAR.

The time-evolution of the physical properties of the BAL were computed by the sea ice thermodynamic model of [46], a refined Semtner 0-layer ice model [47] with detailed snow physics, with the addition of a halodynamic component that describes salinity variations in sea ice (salt entrapment, gravity drainage and flushing) and even more detailed snow physics [25]. A brief mathematical description of the model is provided in section S1 in File S1 (on-line supporting information). The thermodynamic component of the model describes the heat conduction through sea ice and several snow and intermediate snow/ice layers. Given the constant sea ice bulk salinity and the surface and ocean temperatures, brine salinity and volumes can be computed at different depths according to [48], and in particular within the BAL. A simple parameterization based on the Bouguer-Lambert law [49] was used for the computation of the Photosynthetically Active Radiation (PAR) penetrating and reaching the BAL (see File S1). The BAL thickness increases as long as sea ice grows to a maximum of about 0.11 m, then it decreases similarly to sea ice until the end of the ice season (Fig. 5A). The relative brine volume of the BAL decreases until a minimum of about 6.5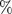 (Fig. 5B), larger than 5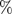 by definition, while brines and sea ice temperatures decrease (Fig. 5C) and brine salinity increases (Fig. 5D). The brine temperature is close to the freezing point of seawater and does not go below −2.5°C, suggesting no temperature limitation to algal growth. The brines salinity range is also small, between 32 and 46, indicating no regulating role of salinity. Finally, the amount of PAR that reaches the BAL continuously decreases to very low values until snow melting, despite the increase in surface irradiance, pointing to a potential limiting role of light. As snow begins melting, the amount of PAR increases exponentially (Fig. 5E).

**Figure 6:**
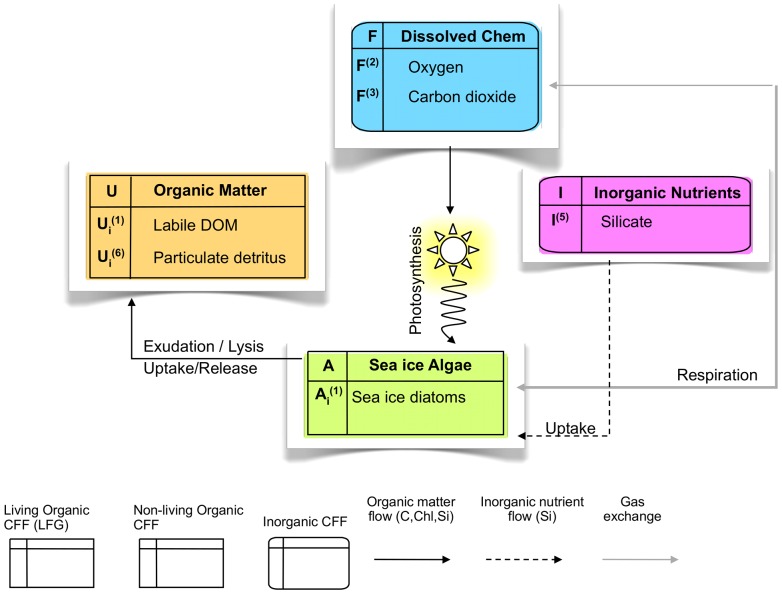
Scheme of the intermediate complexity sea ice biogeochemical model presented in this work. The model includes: 3 inorganic Chemical Functional Families (CFF) i.e. 2 gases (F, oxygen and carbon dioxide) in dissolved forms and 1 macronutrient (I, silicate); 1 non-living CFF encompassing dissolved and particulate organic matter (U); and 1 living CFF of sea ice algae (A), i.e. diatoms. Organic matter flows are due to photosynthesis of sea ice algae and to exudation, lysis, uptake and release of DOM and POM. Inorganic nutrient flows is the silica uptake. Gas exchange is due to oxygen production and carbon dioxide consumption by sea ice algae.

We also choose an intermediate approach for the ecosystem complexity, simplifying the comprehensive model used in [25] that requires a large number of initialization and validation data that are yet far to be available in all sea ice observational sites. In a stoichiometrically-flexible network the model of [25] describes inorganic nutrients (NO_3_+NO_2_, NH_4_, PO_4_, SiO_4_), 2 functional groups of algae (adapted and survivors), particulate and dissolved organic and inorganic matter, and gases such as carbon dioxide and oxygen, for a total number of 22 state variables. The simplified version of the model presented here features one single limiting macronutrient (SiO_4_) and one single group of sea ice algae, i.e. diatoms, generally dominant in the sea ice habitat [50], detritus and gases for totally 9 state variables. A schematic diagram of the model is presented in Fig. 6, model's variables and parameters are reported in Table S2 and Table S3 in File S1, while a mathematical description of the model is given in section S2 in File S1. The limiting nutrient is silicate, but any other nutrient can be chosen as model's currency. Silicon was chosen because the functional group of algae is made of diatoms that require silicate uptake. If the model must have one single chemical component as currency, then silicon is likely to be the most appropriate for the sea ice system. However, many oceanic models use nitrogen as model's currency since it often the most limiting in the oceans. In this latter case, modellers can choose if either increasing the number of state of variables of their model including both silicon and nitrogen components, either if using a N:Si conversion factor. Silicate dynamics differentiate from nitrate and phosphate dynamics as silicate does not accumulate in the cell and it is more likely to be parameterized with a simple Michaelis-Menten function (e.g [51]) and thus directly controls carbon photosynthesis. If nitrate or phosphate are instead chosen as most limiting nutrient, those are decoupled from carbon uptake because of the existence of cellular storage capabilities. The co-limitation from all nutrients can be done with a threshold method, as in [25], and it is considered in the parameterization of some processes such as chlorophyll synthesis and sinking. Multiple nutrient limitation is different for nutrients that can be stored in the cell (nitrate and phosphate) and nutrients that cannot (silicate). [25] allows three alternative ways to combine N and P limitation: the minimum among the two nutrients, a threshold combination (Liebig-like) and a multiplicative approach [51].

[28] showed that sea ice diatoms need to be both photoadapted and photoacclimated to the sea ice light environment. The same optimum Chl:C ratio for sea ice diatoms (0.03 mg Chl/mg C) is thus kept here, able to change according to the organisms' requirements. The simplified model is thus the same as that of [28], but it is characterized by only three basic constituents (C, Chl and Si) and by the physiological rates of photosynthesis, respiration, mortality/excretion and nutrient uptake as presented in Fig. 6. A comparison between this simplified version of the model and the standard and more comprehensive model of [25] is highlighted in the next section.

We coupled the sea ice model with a simple slab ocean, which is meant to represent the mixed layer depth under sea ice (15 m). We defined in seawater the same constituents and processes that we find in sea ice with the addition of bacteria and microzooplankton, as in [28]. The ocean model was initialized according to typical winter mixing conditions: 8.0 mmol Si m^−3^ of dissolved silicate and 1 mg C m^−3^ of diatoms in seawater. The sea ice model did not need to be initialized because it is controlled by the exchanges of dissolved and particulate matter between sea ice and seawater. As a coupling method between the ocean and the sea ice habitat we choose again a method of intermediate complexity [25], [41], which defines the fluxes at the interface as a function of sea ice growth/melt velocities and thickness of the BAL. A complete mathematical description of the coupling fluxes are given in section S2 in File S1 (on-line supporting information).

### Reference simulation

**Figure 7:**
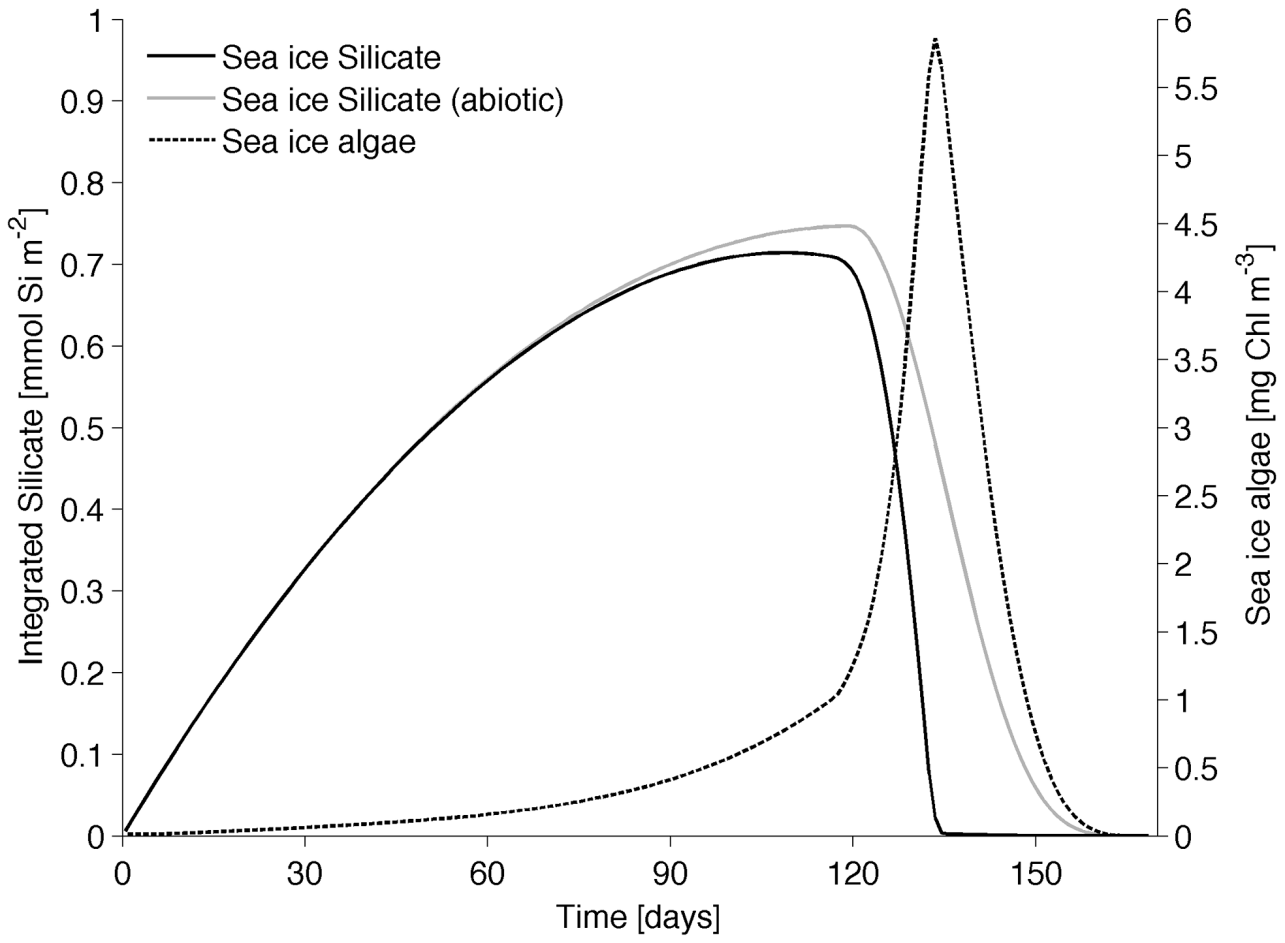
Reference simulation for the typical season of first-year ice in the low Arctic. Vertically integrated dissolved silica concentration in sea ice for the reference and abiotic simulations and Chl volume concentration of sea ice algae in the BAL. Silicate concentration is presented with the integrated value to show the progressive increase of nutrients in the BAL.

The reference simulation (S0) reproduces an enrichment of dissolved silica during the sea ice growth season, followed by a sharp decrease due to the combined action of silica uptake by algae and brine loss due to melting (Fig. 7). The algae bloom reaches its peak at day 134 when nutrients are about to be exhausted (Fig. 7) and the eventual depletion is the combined results of nutrient utilization and volume loss in the biological layer. To show the coexistence of growth and habitat loss processes we also show the silicate curve obtained with a simulation that does not include biological uptake (abiotic simulation, Fig. 7). The difference between the curves represents the amount of nutrient used for sea ice algal growth.

**Figure 8:**
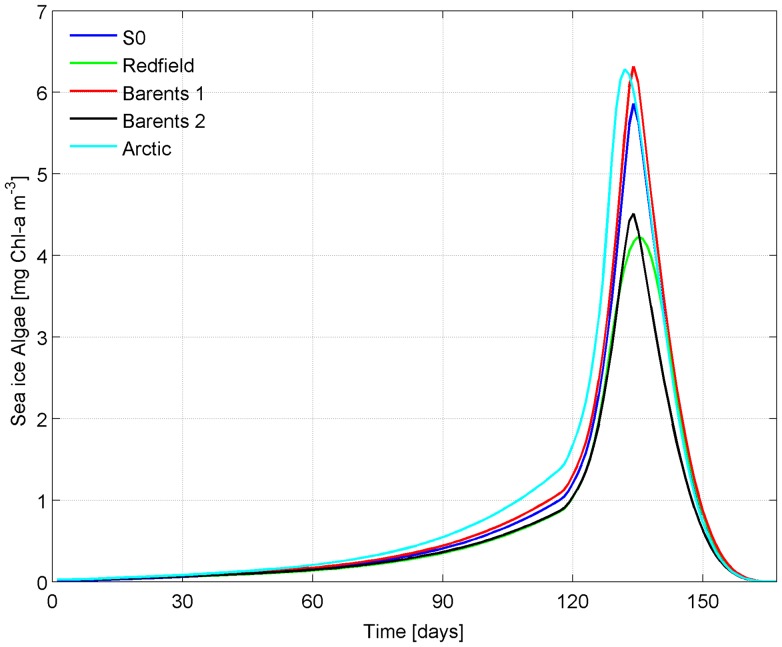
Chl comparison between the standard model of[25] and the simplified model described in this work. Chl comparison between the normal idealized case (S0) produced by the simplified model presented in this work, and by the more comprehensive model of [25] when nutrients are initialized: i) with Redfield ratio with respect to silicate (15 Si: 16 N: 1 P), ii) with non-Redfield values reported from the Barents Sea (Barents 1 = 8 mmol Si 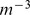; Barents 2 = 6 mmol Si 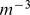), and iii) using average values reported for the entire Arctic Ocean.

The potential error that is made by using this simplified model rather than the more comprehensive model of [25] can be estimated by comparing results given for S0 by both models (Fig. 8). As the model of [25] requires a larger set of variables to be initialized (nitrate, phosphate and survivor algae), several values were considered. The same initial concentration in seawater as for sea ice diatoms was given to survivor algae (1 mg C m^−3^), while for nutrients initialization we compared: (i) a Redfield-like initialization 15 Si: 16 C: 1 P given 8 mmol m^−3^ of initial silicate as in S0; (ii) non-Redfield typical concentrations of the open Barents Sea [52], with silicate ranging between 6 and 8 mmol m^−3^, 12 mmol m^−3^ of nitrate and 0.85 mmol m^−3^ of phosphate (iii) average concentrations reported for the whole Arctic in the Hydrochemical Atlas of the Arctic Ocean [53] (13.20 mmol m^−3^ of silicate, 3.28 mmol m^−3^ of nitrate and 0.83 mmol m^−3^ of phosphate). All simulations reproduce a similar bloom timing but different bloom magnitude (Fig. 8): while the “Redfield-like” and “Barents 2” runs have a smaller peak, the “Barents 1” and “Arctic” are more similar to the S0 peak. Changes in the initial pelagic nutrient conditions have more a direct effect on the magnitude of the bloom rather than on its timing as also shown in [28].

### Sea ice bloom indicators

Although the maximum amplitude (*a_m_*) and the time of the peak (*t_m_*) of an algal bloom may be identified with frequent observations, the exact extent is a matter of definition. Time of initiation and termination may be individuated according to a threshold criterion that can be set in absolute terms (concentration) or in relative terms (some fraction of the maximum amplitude, [54]). As for phytoplankton, the relevant phases of ice algae phenology are the time of initial growth *t_i_*, the time of maximum amplitude 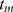 and the duration 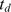. Such kind of ecological indicators are objective-oriented metrics and different methods have been described to investigate phytoplankton phenology. [15] recently studied the Arctic sea ice algal and phytoplankton phenology in terms of maximum amplitude and timing of the peak. However, the usage of indicators of phenological phases, such as time of initiation and duration, in ice algae modelling is yet to start. Considering the large variability in maximum chlorophyll amplitude that can be found in sea ice (e.g. Table 8.1 in [7]), an index that considers a relative threshold rather than an absolute value for indicating the bloom initiation and termination seems more appropriate. Among the potential candidates, we propose to use the anomalies given by the amplitude of the concentration minus its standard deviation. Positive values will thus represent the period of bloom activity in sea ice. This index is independent of the length of the time series (sea ice seasons may present a large variability in the world ocean) and it is less biased by the long period of quiescence that may be observed when light penetration is the limiting factor and the only variability is controlled by the boundary flux with the ocean.

The indices computed from the reference simulation S0 – and from all the other experiments that are following presented – are given in Table 2. The bloom initiation is well at the end of the sea ice season and starts some days after the idealized snow function begins to decrease (day 120). Over a period of about 170 days of sea ice, the bloom period derived from the proposed indicator is a small portion of 26 days. We notice that the bloom indices can be computed as anomalies respect to Chl or carbon content and the results are slightly different due to the parameterization of the natural process of light acclimation. The Chl bloom is longer and starts earlier than the C bloom, implying that there may be a mismatch between indicators computed in terms of biomass or abundances and the ones derived from bulk chlorophyll values.

**Table 2:** Phenology indicators from the reference and scenario simulations.

Simulation	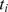	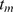	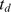	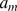
S0: Reference	121(124)	134	26(23)	5.9(335.4)
S1a: Nutrient reduction	121(125)	135	27(23)	2.6(150.9)
S1b: Nutrient increase	124(126)	135	24(22)	11.2(656.8)
S2a: Snow reduction	94(95)	110	40(41)	17.1(596.1)
S2b: Snow increase	134(137)	149(150)	25(21)	1.1 (85.7)
S3a: Early snow melt	121(124)	134	26(23)	5.6(342.3)
S3b: Late snow melt	121(124)	134	26(23)	6.0(330.7)
S4a: 25% shorter ice season	96(98)	106	22(20)	4.3(291.2)
S4b: 50% shorter ice season	70(72)	79	15(14)	2.4(180.2)

The indices are: day of initial growth 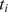, day of maximum amplitude 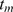, duration 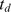 and maximum amplitude 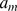 over the bloom duration. Values are referred to the sea ice algae Chl content and in brackets to carbon content. If no value is given in brackets the numbers coincide.

## Results and Discussion: Response to Sea Ice Scenarios

The chosen evolution of sea ice environmental conditions in the Arctic is an idealized case that cannot encompass all the variability that may be observed in reality. We have therefore prepared a set of “scenarios” considering the possible events that may be typically found in this kind of ecosystem to show how a rather simple model can give hints on the underlying real-world processes. The set of sensitivity experiments investigates the dependence on the following parameters:

- S1: change in the pelagic nutrient availability
- S2: variation of snow thickness
- S3: shift in the day of snow melt
- S4: reduction of the length of the ice season

**Figure 9:**
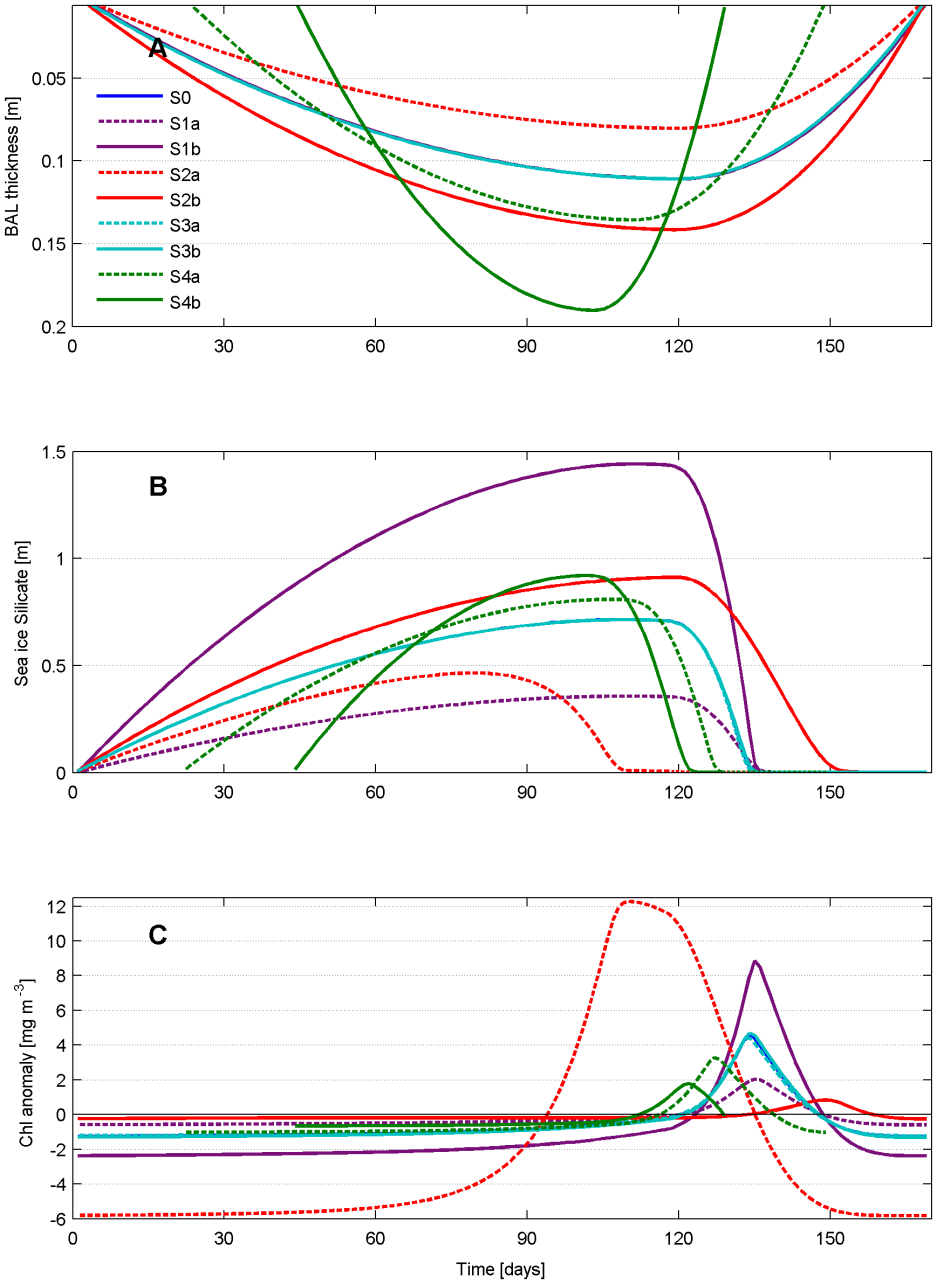
Model response to different scenarios. Temporal evolution of: A) the BAL thickness (note the curves of experiments S0, S1 and S3 coincide); B) silicate concentration (note the curves of experiments S3 coincide); and C) sea ice algae Chl anomalies computed by subtracting the respective standard deviation. Positive values identify the bloom period in each scenario.

The resulting indices of algal phenology for each scenario are reported in Table 2 and a detailed explanation of the model response is given in the next sections. Fig. 9 presents an overview of the changes in the BAL thickness, silicate concentrations and sea ice algae Chl, the latter shown as anomalies from the respective standard deviation. Scenarios S1 and S3 assume no change in the length of the idealized ice season and the BAL remains unchanged from the reference simulation (Fig. 9A). The thickness of the biotic layer is mainly controlled by snow thickness as further analyzed later on. Nutrient concentrations vary in each scenario, except in S3 (Fig. 9B). In S1, silicate is prescribed to be smaller (S1a) and larger (S1b), while in S2 and S4 the dynamics of the nutrient is driven by the fluxes at the ice-ocean boundary in combination with the respective biological processes of uptake and remineralization. The timing and duration of the bloom in each scenario are identified in Fig. 9C using the proposed indicator, where we observe a substantial effect on the bloom features, with the largest impact driven by variations of snow thickness and the length of the ice season.

### Sensitivity to nutrient concentrations

The most simple sensitivity experiment is related to the initial nutrient concentration, which in our case represent the winter background of dissolved silica that may be found in different Arctic regions. We perform such experiment by halving the seawater concentration to a typical value of an open ocean (4 mmol m^−3^, scenario S1a, Fig. 9B) compared to the reference S0 of 8 mmol m^−3^ (Fig. 9B, S0) and by doubling it as it may be found in coastal waters with land-fast ice (16.0 mmol m^−3^, S1b, Fig. 9B). The response of sea ice algae to the variation in silicate concentration is almost linear (Fig. 9C): larger concentrations are associated with larger blooms and viceversa. No effect is seen on the timing of the bloom but only on the amplitude (Table 2). Among all scenarios, the smallest nutrient concentration (Fig. 9B, S1a) results in one of the lowest biomasses (Fig. 9C, S1a) and the highest silicate concentration (Fig. 9B, S1b) among the largest blooms (Fig. 9C, S1b).

### Sensitivity to snow thickness

**Figure 10:**
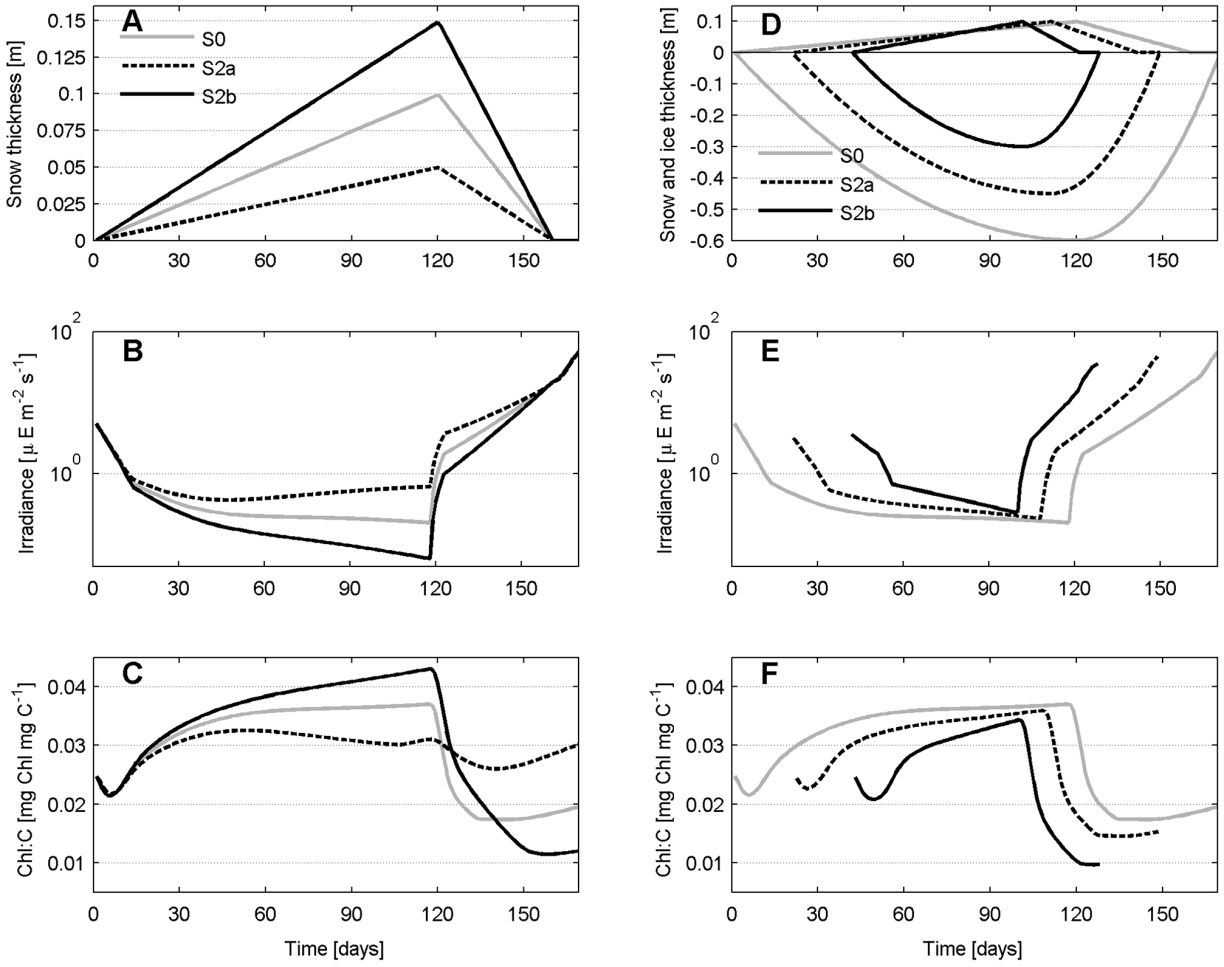
Sensitivity to snow thickness (S2) and season length (S4). Thickness (top), irradiance (middle) and Chl:C ratio (bottom) for S2 (A, B and C) and S4 (D, E and F). Note the log scale in panels B and E.

Light extinction through snow is extremely high (see the supplementary Table 1 in File S1) and a different snow thickness is expected to highly affect the amount of light reaching the bottom sea ice and the response of primary producers. In scenario S2, the initial day of solid deposition, the day of maximum snow cover and the day of complete snow melt are not changed. We only consider a different maximum value of snow thickness, from a minimum of 0.05 m (S2a), to a maximum of 0.15 m (S2b, Fig. 10A). There are large differences both in the timing and the magnitude of the bloom (Fig. 9C), with thinner snow associated to 3-times larger Chl concentration and 24 days earlier bloom (Table 2). Since the bloom starts at day 94 and peaks at day 110 (Table 2), well before snow starts melting (day 120), we conclude that a snow cover of 0.05 m is not light-limiting. This is also confirmed by the temporal dynamics of the algae Chl:C ratio, which is stable during the whole season (Fig. 10C), indicating no specific acclimation needs. This is not true instead in S0 and S2b, where we observe a gradually increasing acclimation to dark conditions (larger Chl:C ratio) until snow melts and later an opposite type of acclimation, i.e. to high light conditions and thus a smaller Chl:C ratio, also in this case more pronounced in S2b than in S0.

### Sensitivity to the day of snow melt

Scenario S3 maintains the maximum snow thickness of 0.10 m as in the reference simulation S0, but varies the day of complete snow melt, shifting it backward to day 150 (S3a) and postponing it to day 170 (S3b). As described in the previous scenario, light attenuation by snow is proportional to the amount of light that reaches the surface and at the same latitude this depends only on the day of the year. The sooner snow completely melts, the sooner larger amount of light penetrates sea ice and reaches its bottom. And the longer the ice season the larger will be the amount of incoming short-wave radiation until the maximum reached at the summer solstice. By changing this parameter we do not observe any significant difference in the peak timing (Table 2, day 134 as in S0) and amplitude (not more than about 5% difference with the maximum amplitude in terms of Chl in S3b, Fig. 9C). We attribute this to the fact that snow cover is light-limiting for most of the ice season, as found in analyzing scenario S2. Even though an earlier snow melt provides the bottom communities with a larger amount of light, there is not enough time for the community to develop further as the sea ice habitat is already shrinking by melting underneath and the scenario presenting a later day of snow melt is still not sufficient to significantly change the bloom characteristics.

### Sensitivity to the length of the ice season

It was pointed out in the Introduction that ice seasons in the Arctic are generally projected to be shorter. We look in scenario S4 at the response of sea ice algae to shorter ice seasons, while keeping the same amount of maximum snow cover (0.10 m) as in the reference simulation S0. We do that by reducing the ice season length of an equal amount of days at the beginning and at the end of the season. In scenario 4a the ice season is shorter of about 25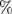 (129 days) and in scenario 4b it is 50 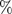 shorter (87 days, Fig. 10D). Accordingly, the ice thickness is also reduced. An important feature of this experiment is the fact that the maximum ice and snow thicknesses are reached earlier in the year, allowing us to analyze how the biota respond to different sunlight availability, depending only on the day of the year rather than on the snow thickness itself, which is 0.10 m in all scenarios. A 25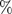 shorter ice season (S4a) produces a 28-days earlier and about 30 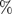 smaller bloom (Table 2 and Fig. 9C). The 50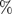 shorter ice season (S4b) shows a similar response, producing an even more accentuated smaller bloom (less than half of the one in S0 (Fig. 9C). The Chl:C ratio (Fig. 10E) clearly shows that in both S4a and S4b the process of light acclimation to darkness is interrupted by the onset of melting snow never reaching the values found in S0, and the shorter is the season the earlier is the interruption. Despite the larger PAR (Fig. 10E), space (Fig. 9A), and nutrient availability (Fig. 9B), the shorter the ice season the smaller the bloom. The restricted time window during which the bloom occurs appears to be the main regulating factor for biomass to have sufficient time to be built.

## Conclusions

The qualitative and quantitative importance of the sea ice biota was shortly reviewed and a general framework to develop a sea ice biogeochemical model from the knowledge on pelagic modelling was presented. We considered different levels of ecosystem model complexity (from simple NPZD models to multiple PFT stoichiometric models) and vertical representation (from single to multi-layer models). This list of options is intended to help the modeller to choose the most appropriate set up under different conditions of applicability. In large-scale simulations and coupled configurations, compromises have to be made and model complexity may be reduced for computational reasons. The applied model will be a compromise between resolving the vertical variability of the sea ice biota, the complexity of the food web, the extent of the spatial and temporal scales and the overall computational constraints. In this work we applied this methodology to build an intermediate complex sea ice biogeochemical model from a previously validated realistic application and using a dynamic single-layer vertical representation together with a simplified plankton functional type model with variable stoichiometry. We coupled the sea ice model to the ocean by computing the boundary fluxes as function of growth/melt velocities and sea ice thickness. We presented a coupling strategy between the bottom sea ice layer and the first ocean level that relies on simple concepts of boundary fluxes and ensures strict mass conservation. We emphasize the importance of developing models that clearly obey to such law.

We used in this work some ecological indicators for sea ice algae commonly used to describe phytoplankton phenology, such as the time of initial growth, the time of maximum amplitude, the maximum amplitude value, and the bloom duration. We proposed a modified criteria to detect the timing of the bloom in terms of initiation and duration, based on arguments that refer to the specific seasonal nature of first-year ice. Ecological indicators are objective-oriented tools that can help quantifying temporal and spatial variations, which are very much needed at this time of rapid changes. More studies to test different indices suitable for sea ice biogeochemical modelling are thus envisaged.

**Figure 11:**
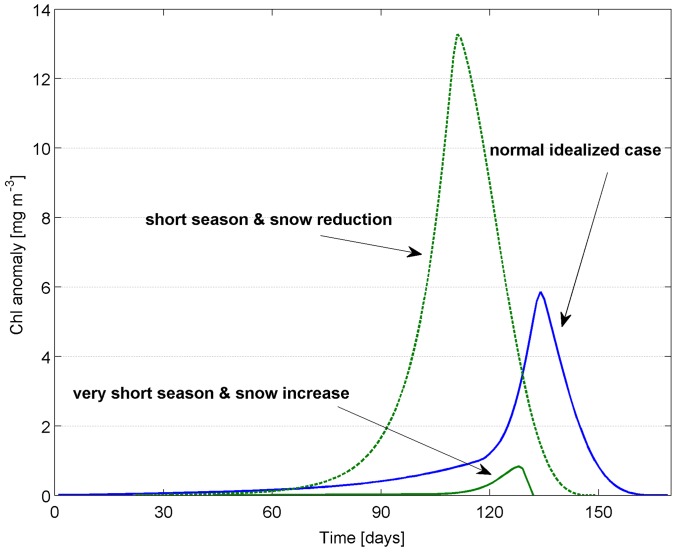
Combined scenarios. Comparison between Chl biomass in the normal idealized case (S0), in a shorter ice season (25% reduction as in S4a) and reduced snow cover (max 5 cm as in S2a), and in a very short ice season (50% reduction as in S4b) and increase snow cover (max 15 cm as in S2b).

Polar regions are rapidly changing and the Arctic is currently facing unpredicted changes. First-year ice is rapidly replacing multi-year ice and our understanding of the changes in primary production patterns in sea ice and in the oceans is mostly a speculative topic [55]. We have used our idealized Arctic ice season not only to show how to develop a sea ice biogeochemical model but also to investigate the relevance of several factors affecting sea ice algae production. Beyond the light limitation due to snow cover and the control exerted by initial nutrients, we showed in this study that the duration of the ice season appears to be an important co-regulating factor. Given the same conditions, excessively short ice seasons would reduce the entire biological community because of lower light availability, as earlier melting dates will occur with fewer hours of sunlight, and because of reduced time to build up biomass. We summarize the studied scenarios and their combination in Fig. 11 (see also figures S1 and S2 in File S1). Favorable conditions such as fewer solid precipitation might counteract the predicted decrease to a certain extent, for instance in the case of a 25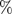 reduction of the sea ice season duration. While the general trend is towards shorter ice seasons, there might be some local variability and some hot spots of productivity might still be found. However, climate projections generally indicate not only shorter ice seasons but also more abundant precipitations in the Arctic [56], thus we can expect that the impoverishment of sea ice would occur at an even faster rate (Fig. 11). We hypothesize that, despite any other positive conditions, excessively short ice seasons would likely reduce the sea ice biological community of producers. Given the same latitude and approximately the same amount of sunlight, this critical condition may be expected to arrive earlier in regions that experience shorter ice seasons. Instead, given the same length of the ice season, this condition is expected to arrive earlier in regions that experience less hours of sunlights.

These considerations are based on the biological production of sea ice diatoms, which we earlier pointed out to be the most abundant and productive group of algae found in sea ice. However, sea ice is inhabited also by other algal groups that have been demonstrated to respond differently to varying light regimes (e.g.[57]). Given enough time, we may either expect the development of new adaptation patterns by diatoms or the dominance of new groups of algae, which should both be accomplished with an increase of model complexity to an extent not considered in the present study.

## Supporting Information

File S1 (PDF)
