# Supplementary material for: Sea Ice Biogeochemistry: A Guide for Modellers
Source: PLoS One. 2014 Feb 25;9(2):e89217. doi: 10.1371/journal.pone.0089217 (PMC3934902; doi:10.1371/journal.pone.0089217)
Supplement: File S1 — (PDF) [file pone.0089217.s001.pdf]

Sea ice biogeochemistry: a guide for modellers
Supporting Information

Letizia Tedesco[1,*], Marcello Vichi[2,3]

1 Marine Research Centre, Finnish Environment Institute, Helsinki, Finland

2 Istituto Nazionale di Geofisica e Vulcanologia, Bologna, Italy

3 Centro Euro-Mediterraneo sui Cambiamenti Climatici, Bologna, Italy

∗ **E-mail: letizia.tedesco@environment.fi**

S0. Model code

The sea ice biogeochemistry model used in this work is part of the BFM package and it is written in FORTRAN90. The model code, physical forcing data, reference simulation and all scenarios discussed in this work are available for download from the BFM web site (http://www.bfm-community.eu). After registration, go to http://bfm-community.eu/download and download the version including the sea ice. Follow the instruction for setting up the pelagic model and compile with the following command: `./bfm_configure.sh -gcd -p STANDALONE_SEAICE`

S1. Equations of the sea ice thermo-halodynamic model used for the forcing computation

This is a selection of the equations of the sea ice halo-thermodynamic model described in [1, 2] that are used in this work to compute the physical forcing data of the Biologically Active Layer (BAL) and that are needed by the biogeochemical model.

For the continuity of the heat conduction at the snow/ice interface [3], we have:

$$K_i \frac{\partial T_i}{\partial z} = K_s \frac{\partial T_s}{\partial z} \tag{1}$$

that can be approximated as:

$$K_i \frac{T_{fr} - T_i}{h_i} = K_s \frac{T_i - T_0}{h_s} \tag{2}$$

where K_i is the sea ice thermal conductivity (2.0 W m K^{-1}), T_{fr} is the seawater freezing temperature (-1.728 ^{o}C), T_i is the temperature at the snow/ice interface, h_i is the sea ice thickness, K_s is the snow thermal conductivity (0.2 W m K^{-1}), T_0 is the surface temperature (prescribed, between T_{fr} and -20 ^{o}C in S0), and h_s is the snow thickness. As the sea ice is continuously in thermal equilibrium, the sea ice bulk temperature (T_{bk}) is always equal to brines temperature (T_{br}) and the latter is related to brines salinity (S_{br}) by the following relationship:

$$T_{br} = -\mu S_{br}, \tag{3}$$

where μ is 0.054 if temperature is in ^{o}C [4]. Brine volume (V_{br}) is related to brine salinity (S_{br}) as:

$$V_{br} = \frac{S_{bk}}{S_{br}} \tag{4}$$

where S_{bk} is the sea ice bulk salinity.

As sea ice is isosaline, we can easily derive the brine temperature and salinity at the boundary with the BAL, considering that the BAL is defined as the sea ice part where the relative brine volume is 0.05:

$$T_{br5} = -\mu \frac{S_{bk}}{0.05}. \tag{5}$$

For the continuity of the heat conduction we will then have:

$$K_i \frac{T_{fr} - T_{br5}}{h_{BAL}} = K_i \frac{T_{br5} - T_i}{(h_i - h_{BAL})} \tag{6}$$

to derive the thickness of the BAL. Assuming the same continuity of heat conduction:

$$K_i \frac{T_{fr} - T_{BAL}}{h_{BAL}/2} = K_i \frac{T_{BAL} - T_i}{(h_i - h_{BAL}/2)} \tag{7}$$

we are able to calculate the ice/brines temperature within the BAL (T_{BAL}). And for the same thermal equilibrium the BAL brine salinity (S_{brBAL}) and relative brine volume (V_{BAL}) within the BAL can be

known:

$$S_{brBAL} = -\frac{T_{BAL}}{\mu} \tag{8}$$

$$V_{BAL} = \frac{S_{brBAL}}{S_{bk}}. \tag{9}$$

The remaining physical variable that is needed is the average amount of Photosynthetically Available Radiation (PAR) found in the BAL. We use a simple parameterization based on the Bouguer-Lambert law (e.g. [5]) to calculate the amount of solar radiation penetrating below the surface:

$$q_x(z,t) = I_0(1-\alpha_x)Q_s e^{\kappa_x z} \tag{10}$$

where I_0 is the fraction of the incident irradiance transmitted through the surface, α_x is the albedo either for snow or ice (see Table S1 for values), Q_s is the incoming short-wave radiation (prescribed, as described in the main paper), and κ_x is the extinction coefficient for either snow or ice (see Table S1 for values).

S2. Equations of the simplified sea ice biogeochemistry model and coupling fluxes with the ocean

This section presents the equations of the sea ice biogeochemical model used in this work, a simplified version of the model described in [6]. Model variables are listed in Table S2 and depicted in Fig. 6 of the main paper and parameter values are given in Table S3.

The rate of change of a sea ice constituent like for instance sea ice algae concentration (A_i, expressed in concentration of carbon (c), Chl (l) and silica (s) integrated over the biological layer) can be described as the sum of the rate of change due to the local biogeochemical processes (bio) and the one due to the exchange with the ocean (oce):

$$\frac{dA_i}{dt} = \left.\frac{dA_i}{dt}\right|_{bio} + \left.\frac{dA_i}{dt}\right|_{oce}. \tag{11}$$

The biological time-rate of change for sea ice algae components considers the processes of photosynthesis (gross primary production= *gpp*), respiration (*rsp*), mortality/excretion (lysis =*lys* and exudation

$= exu$), acclimation and silicate uptake (upt):

$$\left.\frac{dA_c}{dt}\right|_{bio} = \left.\frac{dA_c}{dt}\right|_{F^{(3)}}^{gpp} - \left.\frac{dA_c}{dt}\right|_{F^{(3)}}^{rsp} - \left.\frac{dA_c}{dt}\right|_{U_c^{(1)}}^{exu} - \sum_{j=1,6} \left.\frac{dA_c}{dt}\right|_{U_c^{(j)}}^{lys} \tag{12}$$

$$\left.\frac{dA_l}{dt}\right|_{bio} = \theta_{chl}\left(\left.\frac{dA_c}{dt}\right|_{F^{(3)}}^{gpp} - \left.\frac{dA_c}{dt}\right|_{U_c^{(1)}}^{exu}\right) - \left(\left.\frac{dA_c}{dt}\right|_{F^{(3)}}^{rsp} + \left.\frac{dA_c}{dt}\right|_{U_c^{(6)}}^{lys}\right)\frac{A_l}{A_c} \tag{13}$$

$$\left.\frac{dA_s}{dt}\right|_{bio} = \left.\frac{dA_s}{dt}\right|_{I^{(5)}}^{upt} - \frac{A_s}{A_c}\left.\frac{dA_s}{dt}\right|_{U_c^{(6)}}^{lys}. \tag{14}$$

Gross primary production and respiration involve the uptake and release of dissolved carbon dioxide $F^{(3)}$. Production is controlled by temperature, light and silicate concentration as

$$\left.\frac{dA_c}{dt}\right|_{F^{(3)}}^{gpp} = Q_{10}^{A\,\frac{T-10}{10}} \frac{I^{(5)}}{I^{(5)} + h_A^s} \left[1 - exp\left(-\frac{E_{PAR}}{E_K}\right)\right] r_A^0\, A_c \tag{15}$$

where Q_{10}^A is the characteristic doubling temperature parameter based on the 10 ^{o}C reference temperature, h_A^s is the half-saturation parameter for dissolved silicate uptake, E_{PAR} is the Photosynthetic Available Radiation (PAR) and E_K is the light saturation parameter, the ratio between the realized maximum Chl-a specific photosynthetic rate P_m and the maximum light utilization coefficient (α_A^0):

$$E_K = \frac{P_m}{\alpha} = \frac{r_A^0}{\alpha_A^0}\frac{A_c}{A_l}.$$

Respiration is parameterized as the sum of temperature-controlled basal rate and net growth energy requirement (gross production minus exudation) as

$$\left.\frac{dA_c}{dt}\right|_{F^{(3)}}^{rsp} = Q_{10}^{A\,\frac{T-10}{10}}\, b_A A_c + \gamma_A\left(\left.\frac{dA_c}{dt}\right|_{F^{(3)}}^{gpp} - \left.\frac{dA_c}{dt}\right|_{U_c^{(1)}}^{exu}\right)$$

where b_A is a constant specific rate of respiration and γ_A is a fraction of the assimilated production. The exudation losses are also linearly proportional to gross primary production

$$\left.\frac{dA_c}{dt}\right|_{U_c^{(1)}}^{exu} = \beta_A \left.\frac{dA_c}{dt}\right|_{F^{(3)}}^{gpp}. \tag{16}$$

Cell mortality in sea ice algae is parameterized as a first order process dependent on the internal

physiological state (the nutrient quota) and a constant maximum specific lysis rate d_A^0. Since in this case the only limiting nutrient is silicate which is not stored internally in the cell, the term is simplified to

$$\sum_{j=1,6} \left.\frac{dA_c}{dt}\right|_{U_c^{(j)}}^{lys} = d_A^0 A_c. \tag{17}$$

Net Chl-a synthesis is a function of acclimation to light conditions, nutrient availability and turnover rate. The rate of change of net photosynthesis in (13) is controlled by the dynamical Chl:C ratio θ_{chl} originally proposed by [7], which regulates the amount of Chl-a in the cell according to a non-dimensional ratio between the realized photosynthetic rate in (15) and the maximum potential photosynthesis:

$$\theta_{chl} = \theta_{chl}^0 \frac{\left[1 - exp\left(-\frac{E_{PAR}}{E_K}\right)\right] r_A^0 A_c}{\alpha_A^0 E_{PAR} A_l} \tag{18}$$

where θ_{chl}^0 is the maximum quotum of Chl-a:C.

Silicate uptake for sea ice diatoms in (14) is also derived from pelagic diatoms, linearly coupling net carbon growth

$$G_A = \left.\frac{dA_c}{dt}\right|_{F^{(3)}}^{gpp} - \left.\frac{dA_c}{dt}\right|_{U_c^{(1)}}^{exu} - \left.\frac{dA_c}{dt}\right|_{F^{(3)}}^{rsp} - \left.\frac{dA_c}{dt}\right|_{U_c^{(1)},U_c^{(6)}}^{lys} \tag{19}$$

by means of an optimal cellular quota Si:C s_A^{opt} as

$$\left.\frac{dA_s}{dt}\right|_{I^{(5)}}^{upt} = \max\left(0,\, s_A^{opt} G_A\right). \tag{20}$$

The rate of biogenic silica release in (14) is directly derived from the carbon-based mortality rate (17).

The exchange rate with the ocean in eq. (11) has to be carefully specify to ensure mass conservation with pelagic constituents. When an ice-free ocean is freezing, the first term on the right-hand side of eq. (11) will be zero and the initial concentration of sea ice algae will only be due to the ocean boundary flux with the variable concentration in seawater. Let X_{si} and X_{sw} be the concentration of a generic variable in sea ice and sea water, then the flux can be written as a function of sea ice growth velocity v_{si}:

$$\left.\frac{dX_{si}}{dt}\right|_{X_{sw}}^{oce} = \frac{X_{sw} * v_{si}}{h_{si}} \tag{21}$$

where h_{si} is the thickness of the growing sea ice where biology can be found. Note that the thickness parameter depends on the choice of the model vertical layout and thus h_{si} can be either constant or the variable thickness of the layer in contact with the ocean. This boundary flux is assumed to exist over the entire growth season. At the end of the ice season the flux is generally reverted as there will be a certain amount of X_{si} left in sea ice that will be transferred to seawater. The concentration loss from melting sea ice, which is equivalent to the concentration growth in seawater may be written similarly to (21):

$$\left.\frac{dX_{sw}}{dt}\right|_{X_{si}}^{oce} = \frac{X_{si} * v_{si}}{h_{sw}}. \tag{22}$$

A further note must be added when integrated sea ice concentration is used. The equation will return a concentrations of X in mmol m^{-3} that will be added at the first level of the ocean. In the case of the Biologically Active Layer used in this work we specify the velocity of the boundary fluxes in eq. (21) and eq. (22) as a function of the growth/melt rate $\frac{dh_i}{dt}$ [6, 8]. The rate of change of dissolved silica is then:

$$\frac{dI^{(5)}}{dt} = \left.\frac{dA_s}{dt}\right|_{I^{(5)}}^{upt} + \left.\frac{dI^{(5)}}{dt}\right|_{N^{(5)}}^{oce} \tag{23}$$

where $N^{(5)}$ is the silicate concentration in seawater, and A_s is the content of silica in sea ice diatoms computed by the eq. (20). The silicate boundary flux is written as:

$$\left.\frac{dI^{(5)}}{dt}\right|_{N^{(5)}}^{oce} = \max\left(0, \frac{dh_i}{dt}\right) \max\left(0,\ N^{(5)} - \frac{I^{(5)}}{h_{BAL}}\right) + \min\left(0, \frac{dh_i}{dt}\right) \frac{I^{(5)}}{h_{BAL}}. \tag{24}$$

At sea ice formation, the uptake term in eq. 23 will be zero and the initial concentration of silicate in sea ice will only be due to the boundary flux with the ocean, i.e.:

$$\left.\frac{dI^{(5)}}{dt}\right|_{N^{(5)}}^{oce} = \max\left(0, \frac{dh_i}{dt}\right) \max\left(0,\ N^{(5)}\right). \tag{25}$$

The boundary flux is positive as long as sea ice grows, and negative as sea ice melts. At the very end of the ice season, the concentration of dissolved silica left in sea ice will be transferred to seawater silicate

as:

$$\left.\frac{dI^{(5)}}{dt}\right|_{N^{(5)}}^{oce} = \min\left(0, \frac{dh_i}{dt}\right) \frac{I^{(5)}}{h_{BAL}}. \tag{26}$$

The sea ice enrichment in living and non living particulate organic matter differs from that of dissolved components as it is only a function of the seawater concentration, the ice growth rate and the actual available space in the sea ice matrix, i.e. the relative brine volume, V_{BAL}. Thus the corresponding boundary flux for e.g. the biogenic silica content of sea ice algae is:

$$\left.\frac{dA_s}{dt}\right|_{P}^{oce} = \max\left(0, \frac{dh_i}{dt}\right) P_s V_{BAL} + \min\left(0, \frac{dh_i}{dt}\right) \frac{A_s}{h_{BAL}} \tag{27}$$

and similar considerations as done in eq. 25 and eq. 26 for dissolved silicate are done for the particulate forms.

References

1. Tedesco L, Vichi M, Haapala J, Stipa T (2009) An enhanced sea ice thermodynamic model applied to the Baltic Sea. Boreal Environ Res 14: 68-80.

2. Tedesco L, Vichi M, Haapala J, Stipa T (2010) A dynamic Biologically Active Layer for numerical studies of the sea ice ecosystem. Ocean Modelling 35: 89-104.

3. Parkinson CL, Washington WM (1979) A large scale numerical model of sea ice. Journal of Geophysical Research 84: 322–3337.

4. Assur A (1958) Composition of sea ice and its tensile strength. In: Arctic Sea Ice, Washington, DC: National Acad. Sci.- Nat. Res. Council, Publication 598. pp. 106-138.

5. Untersteiner N (1964) Calculations of temperature regime and heat budget of sea ice in the central Arctic. Journal of Geophysical Research 69: 4755-4766.

6. Tedesco L, Vichi M, Thomas DN (2012) Process studies on the ecological coupling between sea ice algae and phytoplankton. Ecological Modelling 226: 120-138.

7. Geider R, MacIntyre H, Kana T (1998) A dynamic regulatory model of phytoplanktonic acclimation to light, nutrients, and temperature. Limnol Oceanogr 43(3): 679694.

8. Tedesco L, Vichi M (2010) BFM-SI: a new implementation of the Biogeochemical Flux Model in sea ice. CMCC Research Papers : pp. 17.

Supplementary Tables

Supplementary Table 1. Physical model parameters. Values are from the model of [2].

Symbol	Name	Value	Unit
α_{sc}	albedo of cold snow	0.75	-
α_{sw}	albedo of warm snow	0.65	-
α_{iw}	albedo of warm sea ice	0.55	-
κ_{sc}	extinction coefficient of cold snow	25	m^{-1}
κ_{sw}	extinction coefficient of warm snow	15	m^{-1}
κ_{i10c}	extinction coefficient of top 0.1 m of cold sea ice	13.8	m^{-1}
κ_{i10w}	extinction coefficient of top 0.1 m of warm sea ice	6.5	m^{-1}
κ_{ic}	extinction coefficient of cold sea ice	1.55	m^{-1}
κ_{iw}	extinction coefficient of warm sea ice	1.45	m^{-1}

Supplementary Table 2. List of selected model state variables used in this work. The subscript i indicates the components, e.g. $A_i^{(1)} \equiv (A_c^{(1)};\ A_s^{(1)};\ A_l^{(1)})$.

Variable	Components	Description	Units
$I^{(5)}$	Si	Silicate in sea ice	mmol Si m^{-2}
$F^{(3)}$	C	Carbon dioxide in sea ice	mg C m^{-2}
A_i	C Si Chl	Sea ice diatoms	mg C m^{-2}, mmol Si m^{-2}, mg Chl-a m^{-2}
$U_c^{(1)}$	C	Dissolved organic carbon	mg C m^{-2}
$U_i^{(6)}$	C Si	Particulate organic detritus	mg C m^{-2}, mmol Si m^{-2}
$N^{(5)}$	Si	Silicate in sea water	mmol Si m^{-3}
P_i	C Si Chl	Diatoms in sea water	mg C m^{-3}, mmol Si m^{-3},mg Chl-a m^{-3}

Supplementary Table 3. Ecological and physiological parameters. Values and description of the model parameters.

Symbol	Value	Description	Units
r_0^A	1.5	Maximum specific photosynthetic rate	d^{-1}
Q_{10}^A	2.0	Characteristic Q_{10} coefficient	-
θ_{chl}^0	0.035	Optimal quotum chl-a:C	mg chl mg C^{-1}
α_{chl}^0	1.8 e^{-3}	Maximum light utilization coefficient	mg C (mg chl)$^{-1}$ $mE^{-1}m^2s$
h_s^A	0.1	Half saturation value for Si-limitation	mmol Si m^{-2}
b_A	0.05	Basal specific respiration rate	d^{-1}
γ_A	0.10	Activity respiration fraction	-
β_A	0.05	Excreted fraction of primary production	-
d_{O_A}	0.1	Maximum specific lysis rate	d^{-1}
s_A^{opt}	0.03	Optimal Si:C ratio in sea ice diatoms	mmol Si mg C^{-1}

Supplementary Figures

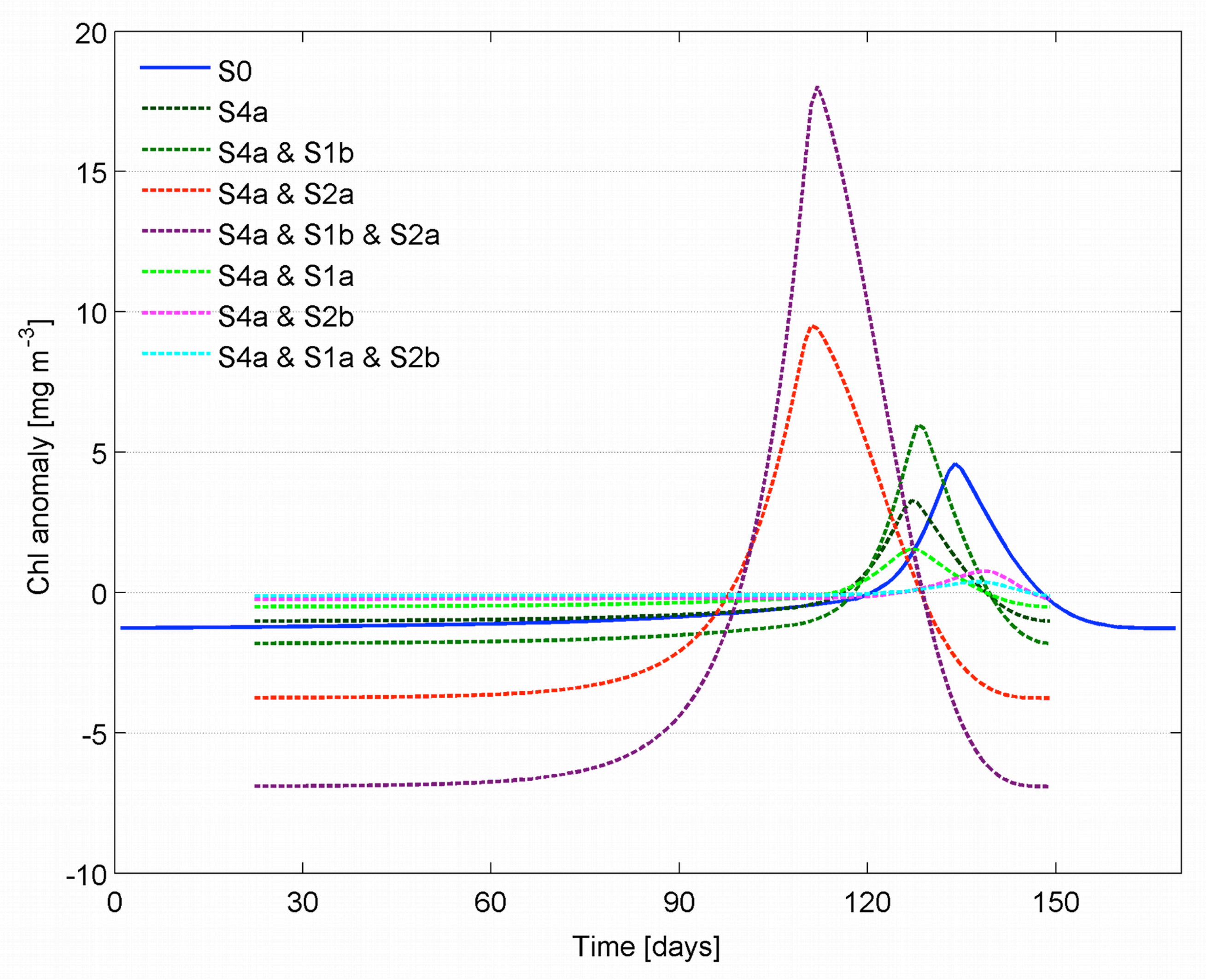

Supplementary Figure 1. Combined scenarios in a short ice season case (S4a). Comparison between the normal idealized case (S0), the short ice season case (25% reduction, S4a), and a combination of scenarios of S4a with decreased nutrients (4 mmol m^{-3} as in S1a), increased nutrients (16 mmol m^{-3} as in S1b), decreased snow cover (5 cm as in S2a), and increased snow cover (15 cm as in S2b). Chl anomalies are computed by subtracting the respective standard deviation.

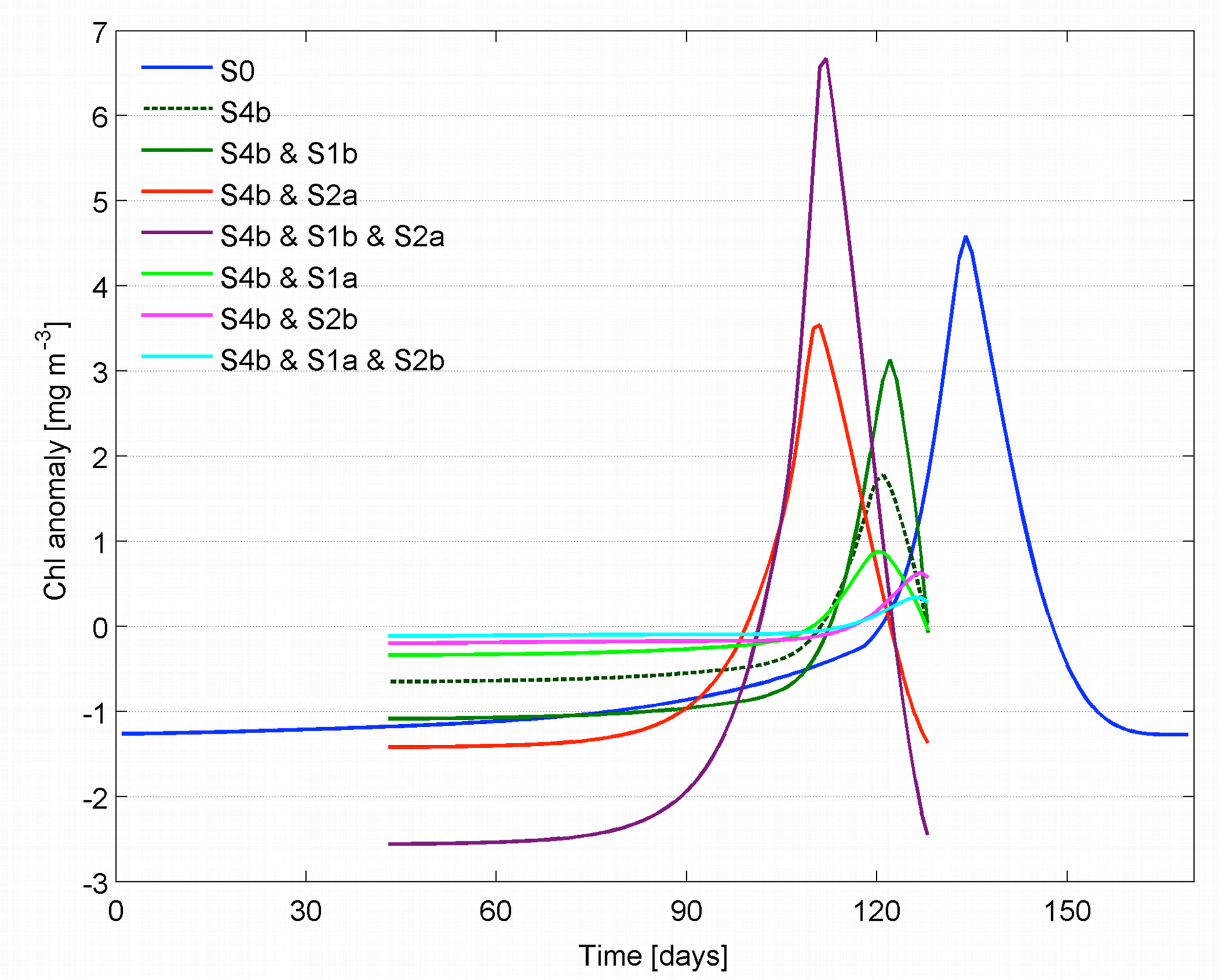

Supplementary Figure 2. Combined scenarios in a very short ice season case (S4b). Comparison between the normal idealized case (S0), the very short ice season case (50% reduction, S4b), and a combination of scenarios of S4b with decreased nutrients (4 mmol m^{-3} as in S1a), increased nutrients (16 mmol m^{-3} as in S1b), decreased snow cover (5 cm as in S2a), and increased snow cover (15 cm as in S2b). Chl anomalies are computed by subtracting the respective standard deviation.
